# Supplementary material for: Perception of Biocontrol Potential of Bacillus inaquosorum KR2-7 against Tomato Fusarium Wilt through Merging Genome Mining with Chemical Analysis
Source: Biology (Basel). 2022 Jan 14;11(1):137. doi: 10.3390/biology11010137 (PMC8773019; doi:10.3390/biology11010137)
Supplement: Supplementary file 1 [file biology-11-00137-s001.zip › biology-1517635-SI.pdf]

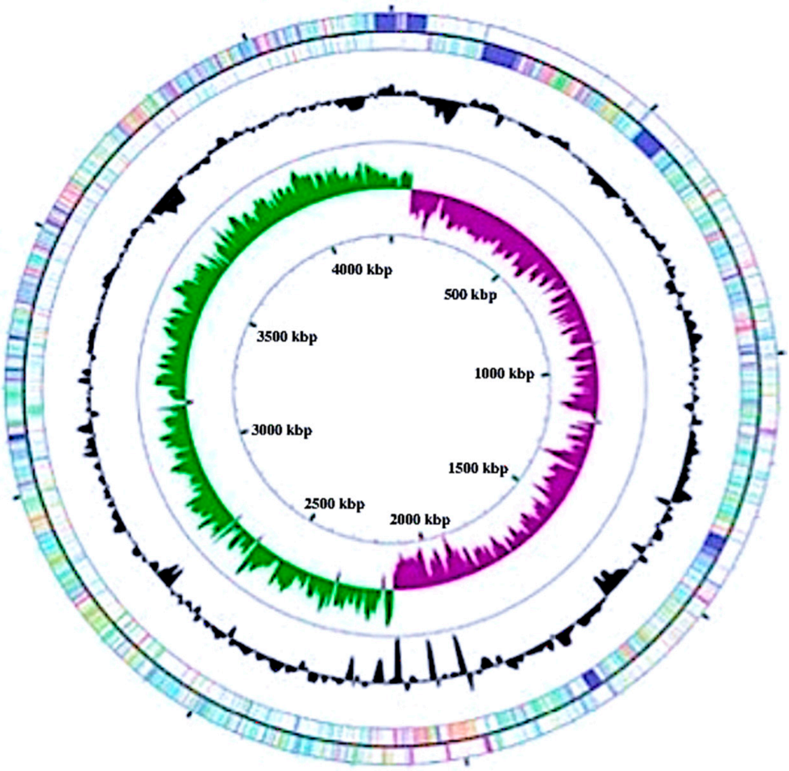

- Chromatin structure and dynamics (1)
- Energy production and conversion (191)
- Cell cycle control, cell division, chromosome partitioning (34)
- Amino acid transport and metabolism (322)
- Nucleotide transport and metabolism (83)
- Carbohydrate transport and metabolism (278)
- Coenzyme transport and metabolism (108)
- Lipid transport and metabolism (103)
- Translation, ribosomal structure, and biogenesis (166)
- Transcription (309)
- Replication, recombination, and repair (150)
- Cell wall/membrane/envelope biogenesis (213)
- Cell motility (44)
- Posttranslational modification, protein turnover, chaperones (104)
- Inorganic ion transport and metabolism (200)
- Secondary metabolites biosynthesis, transport, and catabolism (76)
- Function unknown (913)
- Signal transduction mechanisms (143)
- Intracellular trafficking, secretion, and vesicular transport (26)
- Defense mechanisms (67)
- rRNA
- GC content
- GC skew^{+}
- GC skew^{-}

Figure S1. Circular map of the *B. inaquosorum* KR2-7 genome. Outermost circle (1st): all genes are color-coded according to their functions (see top right); 2nd circle: GC content (black); 3rd circle: GC skew+ (green); 4th circle: GC skew− (violet); 5th circle: scale (bps). GC views were prepared using CGView Server V1.0 (http://wishart.biology.ualberta. ca/cgview/)

Table S1. The comparison of COG functional categories between B. subtilis KR2-7 and B. velezensis FZB42

COG code	Description	KR2-7	FZB42
B	Chromatin structure and dynamics	1	-
C	Energy production and conversion	191	169
D	Cell cycle control, cell division, chromosome partitioning	34	37
E	Amino acid transport and metabolism	322	275
F	Nucleotide transport and metabolism	83	82
G	Carbohydrate transport and metabolism	278	236
H	Coenzyme transport and metabolism	108	120
I	Lipid transport and metabolism	103	122
J	Translation, ribosomal structure and biogenesis	166	155
K	Transcription	309	240
L	Replication, recombination and repair	150	112
M	Cell wall/membrane/envelope biogenesis	213	174
N	Cell motility	44	61
O	Posttranslational modification, protein turnover, chaperones	104	97
P	Inorganic ion transport and metabolism	200	154
Q	Secondary metabolites biosynthesis, transport and catabolism	76	64
R	General function prediction only	-	337
S	Function unknown	913	318
T	Signal transduction mechanisms	143	130
U	Intracellular trafficking, secretion, and vesicular transport	26	169
V	Defense mechanisms	67	-

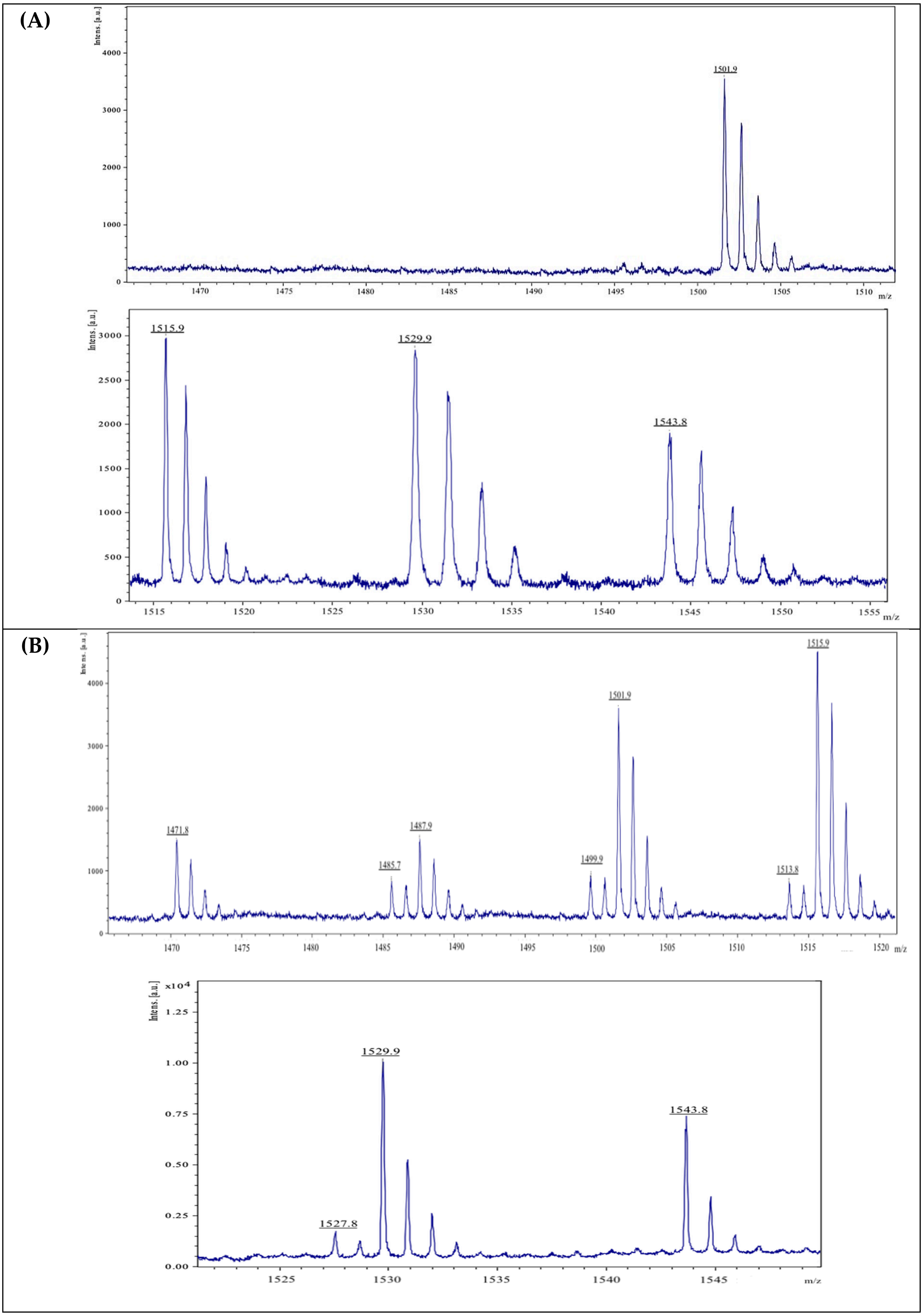

Figure S2. The fengycin mass peaks detected by MALDI-TOF mass spectrometry in A) control cell extract of KR2-7 and B) dual culture cell extract of KR2-7.

(A)

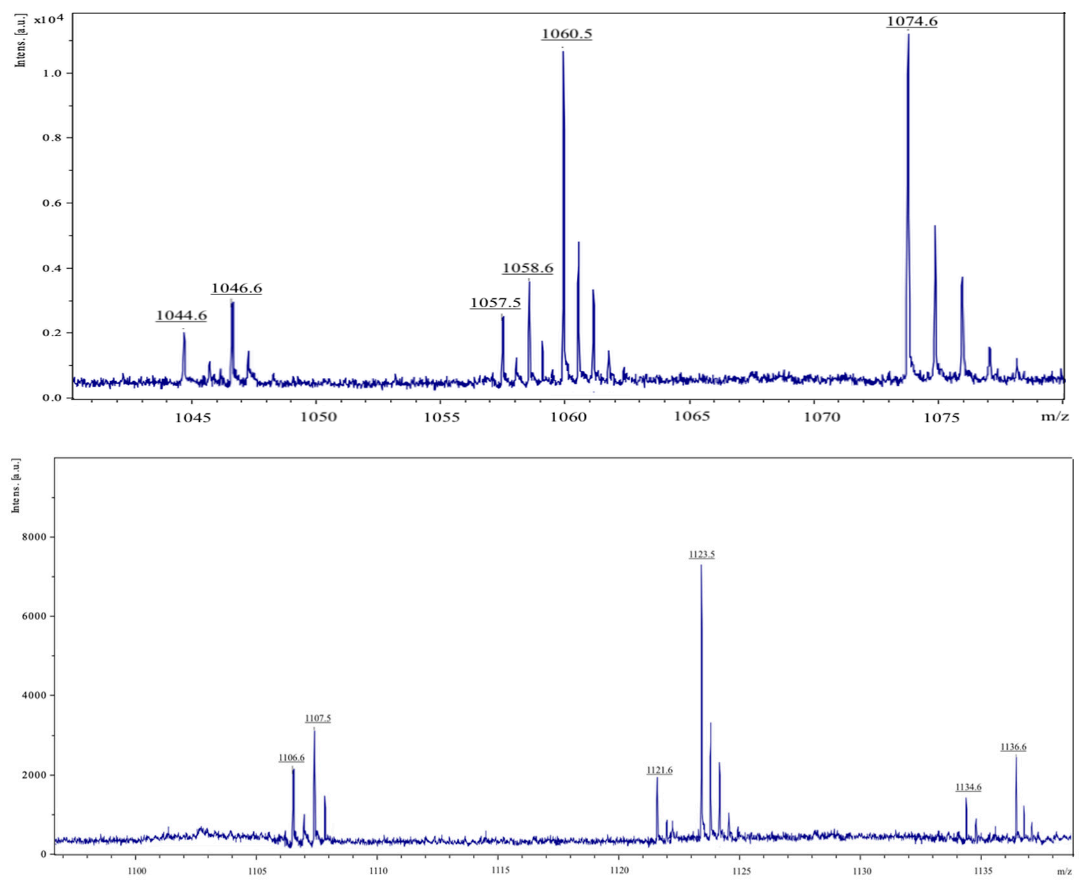

Figure S3. **(A)** The iturin, bacillomycin F, and surfactin mass peaks detected by MALDI-TOF mass spectrometry in A) control cell extract of KR2-7 and B) dual culture cell extract of KR2-7.

(B)

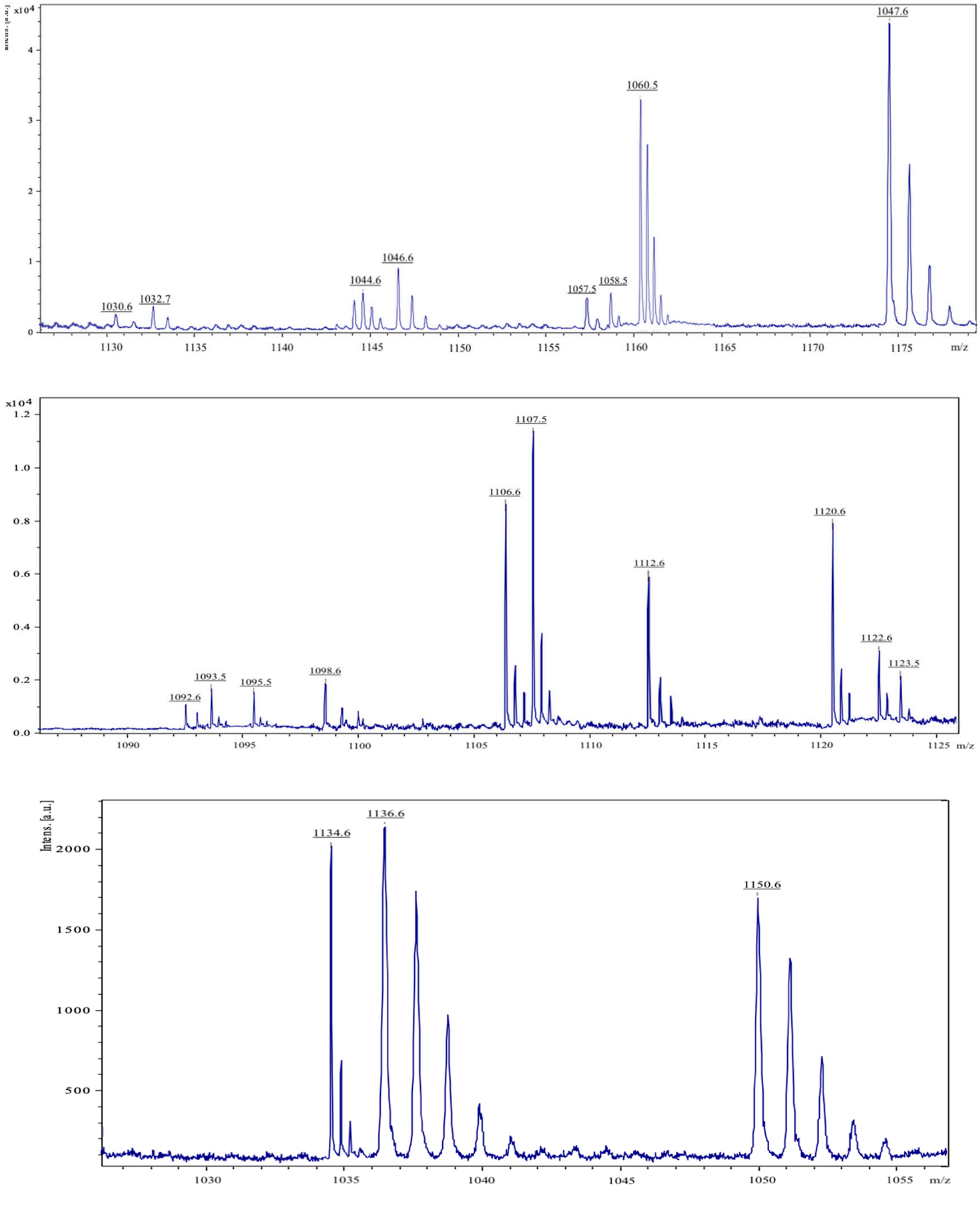

Figure S3. **(B)** The iturin, bacillomycin F, and surfactin mass peaks detected by MALDI-TOF mass spectrometry in A) control cell extract of KR2-7 and B) dual culture cell extract of KR2-7.
